# Carta ao editor referente ao artigo: “Efeitos da infiltração intra-articular de aspirado de medula óssea no tratamento da osteoartrite de joelho: Estudo clínico comparando BMA *versus* corticosteroide e bloqueio genicular”. *Rev Bras Ortop* 2025;60(2):s00451809512. DOI:10.1055/s-0045-1809512

**DOI:** 10.1055/s-0046-1817130

**Published:** 2026-04-28

**Authors:** Rodrigo Monari, Carlos Henrique Pfiffer, Amadeu Davoglio Lorga, Morghana Fin

**Affiliations:** 1 Hospital Santo Antônio, Blumenau, SC, Brasil; 2 Hospital Unimed, Blumenau, SC, Brasil; 3 Clínica Monari, Blumenau, SC, Brasil

Prezado Editor-Chefe da *Revista Brasileira de Ortopedia e Traumatologia* , Dr. Geraldo Motta,

Após a leitura atenta do artigo de Clazzer et al., 1 julgamos pertinente tecer alguns comentários a respeito do delineamento e da interpretação dos resultados apresentados.

1. Aspectos Metodológicos
  O estudo, embora prospectivo e randomizado, apresenta limitações importantes. Houve perda de 15 pacientes ao longo do seguimento, resultando em apenas 35 indivíduos avaliados em 6 meses. Tal redução compromete o poder estatístico originalmente planejado. Além disso, não há detalhamento de cálculo amostral para desfechos clínicos de relevância, tampouco critérios de randomização claramente descritos.
2. Confusão Conceitual sobre a Intervenção
Os autores descrevem o grupo intervenção como tratado com aspirado de medula óssea (BMA, do inglês *bone marrow aspirate* ). Entretanto, conforme a própria metodologia, o BMA foi misturado a dexametasona e injetado intra-articularmente. Esse ponto é crítico: há consenso na literatura de que glicocorticoides exercem efeito deletério sobre células-tronco mesenquimais, reduzindo sua viabilidade, proliferação e diferenciação condrogênica. 2 Tal conduta contraria os princípios da medicina regenerativa e dificulta a atribuição dos resultados ao BMA em si.
3. Interpretação dos Resultados
  Os achados clínicos mostraram diferença significativa apenas no domínio “dor” do Western Ontario and McMaster Universities Arthritis Index (WOMAC) somente após 6 meses, sem melhora significativa em rigidez ou função. Ainda assim, os autores concluem que o BMA representa uma opção terapêutica viável. Consideramos essa afirmação precipitada, pois se baseia em desfechos limitados e em seguimento curto. Infelizmente também observamos que no Instagram da Sociedade Brasileira de Ortopedia e Traumatologia, a conclusão errônea do artigo é que existe melhora da dor e função.
